# Supplementary material for: Identification of PARP-1, Histone H1 and SIRT-1 as New Regulators of Breast Cancer-Related Aromatase Promoter I.3/II
Source: Cells. 2020 Feb 12;9(2):427. doi: 10.3390/cells9020427 (PMC7072628; doi:10.3390/cells9020427)
Supplement: Supplementary file 1 [file cells-09-00427-s001.pdf]

Supplementary Materials

for the manuscript

Identification of PARP-1, histone H1 and SIRT-1 as new regulators of breast cancer-related aromatase promoter I.3/II

Alexander Kaiser [1,2], Thomas Krüger [3], Gabriele Eiselt [1], Joachim Bechler [4], Olaf Kniemeyer [3], Otmar Huber [1] and Martin Schmidt [1,*]

```
         C/EBP 1 (-350/-337)                                   C/EBP 2 (-317/-304)
-350     TTGTTTTGAA  ATTGATTTGG  CTTCAAGGGA  AGAAGATTGC  CTAAACAAAA

                    CRE (-292/-285)                                   SF-1 (-263/-251)
-300     CCTGCTGATG  AAGTCACAAA  ATGACTCCAC  CTCTGGAATG  AGCTTTATTT

         promoter I.3                       exon I.3             CRE (-211/-199)
           TATA box
-250     TCTTATAATT  TGGCAAGAAA  TTTGGCTTTC  AATTGGGAAT  GCACGTCACT

-200     CTACCCACTC  AAGGGCAAGA  TGATAAGGTT  CTATCAGACC  AAGCGTCTAA

                       SF-1 (-136/-124)
-150     AGGAACCTGA  GACTCTACCA  AGGTCAGAAA  TGCTGCAATT  CAAGCCAAAA

-100     GATCTTTCTT  GGGCTTCCTT  GTTTTGACTT  GTAACCATAA  ATTAGTCTTG

                       promoter II TATA box
-50      CCTAAATGTC  TGATCACATT  ATAAAACAGT  AAGTGAATCT  GTACTGTACA

           exon II
+1       GCACCCTCTG  AAGCAACAGG  AGCTATAGAT  GAACCTTTTA  GGGGATTCTG

                                                   coding exon II
+51      TAATTTTTCT  GTCCCTTTGA  TTTCCACAGG  ACTCTAAATT  GCCCCCTCTG

                     translation start
+101     AGGTCAAGGA  ACACAAGATG  GTTTTGGAAA  TGCTGA...
                           M     V  L  E    M
```

Figure S1. SNV in the aromatase promoter I.3/II DNA sequence. The SNV(T-241C) (NC_000015.10:n.51243270T>C; GRCh38.p7 human genome reference) near the promoter I.3 TATA-box is indicated by a bold enlarged letter. Promoter elements are given as reviewed by Chen et al. [7].

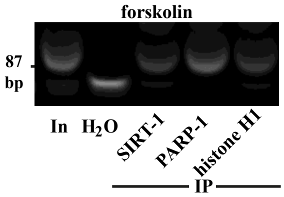

Figure S2. Original gel image for Figure 6E. The gels for analysis of ChIP-experiments with primer set 2 were loaded in another order than the other ChIP-gels. This is the original sample order on the uncut gel.

Table S1. Primers and conditions for PCR amplifications. Cycling conditions used for the amplification of the respective amplicons are given with times and temperatures for each step and number of cycles. Probe numbers refer to the according probe used from the Universal Probe Library (https://lifescience.roche.com/en_de/brands/universal-probe-library.html). Den., denaturation; Ann., annealing; Elo., elongation; Cyc., cycles; F, forward; R, reverse.

Use	Primer		Primer Sequences	Den.	Ann.	Elo.	Cyc.
ChIP	primer-set 1 bp -462 to -197	F	5′- GGCTCTGAGAA GACCTCAACG -3′	15 s/ 94 °C	15 s/ 60 °C	15 x/ 72 °C	40
		R	5′- GTAGAGTGAC GTGCATTCCCA -3′				
	primer-set 2 bp -285 to -199	F	5′- ACAAAATGACTC CACCTCTGGAA -3′	15 s/ 94 °C	15 s/ 60 °C	15 x/ 72 °C	40
		R	5′- AGAGTGACGT GCATTCCCAAT -3′				
Quantification of aromatase mRNA-expression in BAFs	full length aromatase (exon IX-X) (probe 76)	F	5′-CAAACCCAATGA ATTTACTCTTGA-3′	15 s/ 95 °C	1 min/ 60 °C		40
		R	5′-ACCATGGCG ATGTACTTTCC-3′				
	aromatase promoter I.3 (probe 9)	F	5′-CTTGCCTAAATGT CTGATCACATTA-3′	15 s/ 95 °C	1 min/ 60 °C		40
		R	5′-CATGGCTTC AGGCACGAT-3′				
	aromatase promoter II (probe 9)	F	5′-CCCTTTGATTT CCACAGGAC-3′	15 s/ 95 °C	1 min/ 60 °C		40
		R	5′-CATGGCTTC AGGCACGAT-3′				
	GAPDH (human) (probe 60)	F	5′-AGCCACATC GCTCAGACAC-3′	15 s/ 95 °C	1 min/ 60 °C		40
		R	5′-GCCCAATACG ACCAAATCC-3′				
Quantification of aromatase mRNA-expression in transfected PARP-1 wild-type and knock-out MEFs	aromatase exon I.3 5′-region	F	5′- CACGTCACTCTA CCCACTCAAG -3′	15 s/ 95 °C	30 s/ 60 °C	30 s/ 72 °C	45
		R	5′- AGGAACCAGG GCGTATCTCTT -3′				
	aromatase exon I.3 3′-region	F	5′- AGATCTTTCTTG GGCTTCCTTGT -3′	15 s/ 95 °C	30s/ 60°C	30s/ 72°C	45
		R	5′- AGGAACCAGG GCGTATCTCTT -3′				
	firefly luciferase	F	5′- TGGAAGACGCCAA AAACATAAAGAA -3′	15 s/ 95 °C	30 s/ 60 °C	30 s/ 72 °C	45
		R	5′- AGGAACCAGG GCGTATCTCTT -3′				
	GAPDH cDNA (murine)	F	5′- GGTCCCAGCTT AGGTTCATCA -3′	15 s/ 95 °C	30 s/ 60 °C	30 s/ 72 °C	45
		R	5′- CCAATACGGCC AAATCCGTTC -3′				
	GAPDH gDNA (murine)	F	5′-TTGCCGTGAGT GGAGTCATAC-3′	15 s/ 95 °C	30 s/ 60 °C	30 s/ 72 °C	45
		R	5′-TCACTACAGACC				

CATGAGGAGTT-3′

Table S2. MALDI-TOF MS/MS and Nano-ESI-Quadrupol-TOF MS/MS results. Evaluation of ms/ms data was done by Mascot-interface in connection with the NCBI/nr database. All Mascot scores >65 are reliable. Furthermore, manual amino acid sequence alignment was done by protein-BLAST and uniProtkb databases. PARP-1 was identified by MALDI-TOF MS/MS. Histone H1 isoforms were identified by Nano-ESI-Quadrupol-TOF MS/MS. Numbers of identified amino acids / total amino acids and percent identified amino acids are given for each protein. Identified amino acids are indicated in red in the sequences. MW, molecular weight.

Protein	Sequence Coverage	Mascot Score	MW (kDa)	FASTA – Amino Acid Sequence
PARP-1	313/1014 amino acids<br>30.9 %	**646.9**	113	MAEASERLYR VEYAKSGRAS CKKCSESIPK<br>DSLRMAIMVQ SPMFDGKVPH WYHFSCFWKV<br>GHSI**RQPDVE** **VDGFSELRW**D DQQKVKKTAE<br>AGGVAGKGQD GSGGKAE**KTL** **GDFLAEYAKS**<br>**NRSMCKG**CLE KIEKGQMRLS K**KMVDPEKPQ**<br>**LGMIDRW**YHP TCFVKK**RDEL** **GFRPEYSASQ**<br>**LKG**FSLLSAE DKEALKKQLP AIKNEGKRKG<br>DEVDGTDEVA KKKSKKGKDK DSSKLEKALK<br>AQNELIWNIK DELKKACSTN DL**KELLIFNQ**<br>**QQVPSGESAI** **LDRV**ADGMAF GALLPCKECS<br>GQLVFKSDAY YCTGDVTAWT KCMV**KTQNPS**<br>**RK**EWVTPKEF REISYLKKLK VKKQDRIFPP<br>ESSAPAPLAL PLSVTSAPTA VNSSAPADKP<br>LSNMKILTLG KLSQNKDEAK AVIEKLGGKL<br>TGSANKASLC ISTKKEVEKM SKKMEEVKAA<br>NVRVVCEDFL QDVSASTKSL QELLSAHSLS<br>SWGAEVKAEP GEVVAPKGKS AAPSKKSKGA<br>VKEEGVNKSE KRMKLTLKGG AAVDPDSGLE<br>HSAHVLEKGG KVFSATLGLV DIVKGTNSYY<br>**KLQLLEDDKE** **SRY**WIFRSWG RVGTVIGSN**K**<br>**LEQMPSKE**DA VEHFMKLYEE **KTGNAWHSKN**<br>FTKYPKKFYP LEIDYGQDEE AVKKLTVKPG<br>TKSKLPKPVQ ELVGMIFDVE SMK**KALVEYE**<br>**IDLQKM**PLGK LS**RRQIQAAY** **SILSEVQQAV**<br>**SQGSSESQIL** **DLSNRFYTLI** **PHDFGMKK**PP<br>LLNNADSVQA **KVEMLDNLLD** **IEVAYSLLRG**<br>GSDDSSKDPI DVNYEKLKTD I**KVVDRDSEE**<br>**AEVIRK**YV**KN** **THATTHNAYD** **LEVIDIFKI**E<br>REGESQRYKP FRQLHN**RRLL** **WHGSRTTNFA**<br>**GILSQGLRI**A PPEAPVTGYM FGKGIYFADM<br>VSKSANYCHT SQGDPIGLIL LGEVALGNMY<br>ELKHASHISK LPKGKHSVKG LGKTTPDPSA<br>SITLEGVEVP LGTGIPSGVN DTCLLYNEYI<br>VYDIAQVNLK YLLKLKFNFK TSLW
histone H1.1	32/213 amino acids<br>15.0%	**225.5**	31	MSETAPVAQA ASTATEKPAA AKKTKKPAKA<br>AAPRKKPAGP SVSELIVQAV SSSKE**RSGVS**<br>**LAALKKSLAA** **AGYDVEKN**NS RIKLGLKSLV<br>N**KGTLVQTKG** TGAAGSFKLN KKAESKAITT<br>KVSVKAKASG AAKKPKKTAG AAAKKTVKTP<br>KKPKKPAVSK KTSKSPKKPK VVKAKKVAKS<br>PAKAKAVKPK ASKAKVTKPK TPAKPKKAAP KKK
histone H1.2	90/212 amino acids<br>42.4%	**643.2**	31	**MSEAAPAAPA** **AAPPAEKA**PA KKKAAKKPAG<br>VR**RKASGPPV** **SELITKA**VAA SKE**RSGVSLA**<br>**ALKKALAAAG** **YDVEKN**NSRI KLGLKSLVS**K**<br>**GILVQTKGTG** **ASGSFKLNKK** AASGEAKPQA<br>KKAGAAKAKK PAGAAKKPKK ATGAATPKKA<br>AKKTPK**KAKK** **PAAAAVTKK**V AKSPKKAKVT<br>KPKKVKSASK AVKPKAAKPK VAKAKKVAAK KK
histone H1.3	92/221 amino acids<br>41.6%	**734.6**	32	**MSETAPAAPA** **APAPVEKT**PV KKKAKKTGAA<br>AGK**RKASGPP** **VSELITKA**VA ASKE**RSGVSL**<br>**AALKKALAAA** **GYDVEKNNSR** **I**KLGLKSLVS<br>**KGTLVQTKGT** **GASGSFKLNK** **K**AASGEAKPK<br>AKKAGAAKAK KPAGAAKKP**K** **KATGAATPKK**<br>TAKKTPKKAK KPAAAAGAKK VSKSPKKVKA<br>AKPKKAAKSP AKAKAPKAKA SKPKASKPKA<br>TKAKKAAPRKK

histone H1.4	86/219<br>amino acids<br><br>39.3%	**736.1**	32	**MSETAPAAPA APAPAEKT**PV KKKARKAAGG<br>AK**RKTSGPPV SELITKA**VAA SKE**RSGVSLA**<br>**ALKKALAAAG YDVEKNNSRI** KLGL**KSLVSK**<br>**GTLVQTKGTG ASGSFKLNKK** AASGEAKPKA<br>KRAGAAKAKK PAGAAKKPKK AAGTATAKKS<br>TKKTPKKAKK PAAAAGAKKA KSPKKAKATK<br>AKKAPKSPAK AKTVKPKAAK PKTSKPKAAK PKKTAAKKK
histone H1.5	112/223<br>amino acids<br><br>50.2%	**734.6**	32	**MSETAPAETA APAPVEKS**PA KKKTTKKAGA<br>AK**RKATGPPV SELITKA**VSA S**KERGGVSLP**<br>**ALKKALAAGG YDVEKNNSRI** KLGL**KSLVSK**<br>**GTLVQTKGTG ASGSFKLNKK** AASGEAKPKA<br>KKTGAA**KAKK PAGATPKK**PK KTAGAKKTVK<br>KTPK**KAKKPA AAGVKK**VAKS PKKAKAAAKP<br>KKAAKSPAKP KAVKSKASKP KVTKPKTAKP<br>KAAKAKKAVS KKK
